# CCL3L3-null status is associated with susceptibility to systemic lupus erythematosus

**DOI:** 10.1038/s41598-021-98531-6

**Published:** 2021-09-27

**Authors:** Young-Ho Kim, Eunyoung Emily Lee, Hye-Won Sim, Eun-Kyung Kang, Yoon-Ho Won, Dong-eun Lee, Kyeong-Man Hong, Yeong-Wook Song

**Affiliations:** 1 Research Institute, National Cancer Center, Goyang, Gyeonggi-do Republic of Korea; 2 Division of Rheumatology, Department of Internal Medicine, Uijeongbu Eulji Medical Center, Eulji University School of Medicine, Uijeonbu, Gyeonggi-do Republic of Korea; 3 Biostatistics Collaboration Team, Research Core Center, Research Institute, National Cancer Center, Goyang, Gyeonggi-do Republic of Korea; 4 Division of Rheumatology, Department of Internal Medicine, Seoul National University Hospital, Seoul, Republic of Korea; 5 Medical Research Center, Institute of Human-Environment Interface Biology, Seoul National University, Seoul, Republic of Korea

**Keywords:** Immunology, Rheumatology

## Abstract

The correlation between copy number variation (CNV) and the susceptibility to systemic lupus erythematosus (SLE) has been reported for various immunity-related genes. However, the contribution of CNVs to SLE susceptibility awaits more investigation. To evaluate the copy numbers in immunity-related genes such as *TNFAIP3*, *TNIP1*, *IL12B*, *TBX21* (*T-bet*), *TLR7*, *C4A*, *C4B*, *CCL3L1,* and *CCL3L3*, the modified real competitive polymerase chain reaction (mrcPCR) assay was employed, and the association between the copy numbers and SLE susceptibility was analyzed in 334 SLE patients and 338 controls. *CCL3L3*-null status was significantly associated with SLE susceptibility (OR > 18, *P* < 0.0001), which remained significant by Bonferroni’s correction (corrected *P* = 0.0007). However, the significant association between *C4B* low-copy status and SLE susceptibility (OR = 1.6051, *P* = 0.0331) became non-significant by Bonferroni’s correction (corrected *P* = 0.3938). Except for these results, no other significant association between SLE susceptibility and copy number status in other genes was observed. The *CCL3L3*-null status may be a significant factor for SLE susceptibility.

## Introduction

Systemic lupus erythematosus (SLE) is an autoimmune disease predominantly affecting females. In SLE, the uncontrolled production of autoantibodies that react with self-nuclear and cytoplasmic antigens leads to the release of inflammatory mediators and ultimately, to multiple organ damage. The derangement of immune T-cell tolerance has been suggested as a key mechanism of SLE pathophysiology. Even though both genetic and environmental factors have been reported as important, the etiology remains unclear. Major histocompatibility complex (MHC) genes have been the main focus of genetic etiology studies^1^, but non-MHC susceptibility loci have also been recognized as important players, and genome-wide association studies have identified important loci or single nucleotide polymorphisms (SNPs)^2^. Among the important genetic etiological factors, copy number variations (CNVs) in many immunity-related genes have been reported to be associated with SLE. However, the contribution of CNVs in immunity-related genes to SLE susceptibility awaits more investigation.

The correlation of CNVs in various immune-related genes including *IL12B* (GeneID 3593)^3^, *TBX21* (GeneID 30009)^3^, *TLR7* (GeneID 51284)^4^, and *CCL3L1* (geneID: 6349)^5^, in addition to MHC class III genes such as *C4A* (geneID: 720) and *C4B* (geneID: 721)^6,7^ with SLE susceptibility has been reported. *IL-12B* and *TBX21* (or *T-bet*) are Th1 cell-related cytokine and Th1 lineage-specific transcription factor, respectively, and Th1 cells have implications in the pathogenesis of autoimmunity^8^. A higher dosage of *TLR7* is related to disease severity in lupus-prone mice^9,10^, and *TLR7* overexpression induced systemic autoimmunity even in non-lupus-prone mice^11^. Complement component 4 is an effector protein of the immune system, and its total deficiency is one of the strongest genetic risk factors for human SLE^7,12^. *CCL3L1* and *CCL3L3* are *CCL3*-related homologous chemokine genes, and individuals with more *CCL3L1* copies are less susceptible to human immunodeficiency virus (HIV) infection and a deviation from the average copy number (CN) of *CCL3L1* was related to higher susceptibility to SLE^5^ or rheumatoid arthritis^13^. Therefore, evaluating the association of CNVs in these genes with SLE susceptibility in a cohort of 334 patients and 338 controls would give more insight into the genetic etiology of SLE.

*TNIP1* (geneID: 10318) has been reported among the highest-scoring non-MHC genes across multiple genome-wide association studies (GWAS) in many autoimmune diseases including SLE^14–16^. Another one of the highest scoring loci in the non-MHC locus is *TNFAIP3* (geneID: 7128), which is associated with various autoimmune diseases including SLE^17–19^. Although the association of CNVs in *TNFAIP3* and *TNIP1* with SLE susceptibility has not been reported, associations with RA susceptibility have been reported^20^. Therefore, the contribution of CNs in *TNFAIP3* and *TNIP1* in SLE susceptibility was also evaluated in the present study. To evaluate the CNs, the modified real competitive polymerase chain reaction (mrcPCR) method was employed.

## Results

### Establishment of mrcPCR assays for CN determination

**Figure 1:**
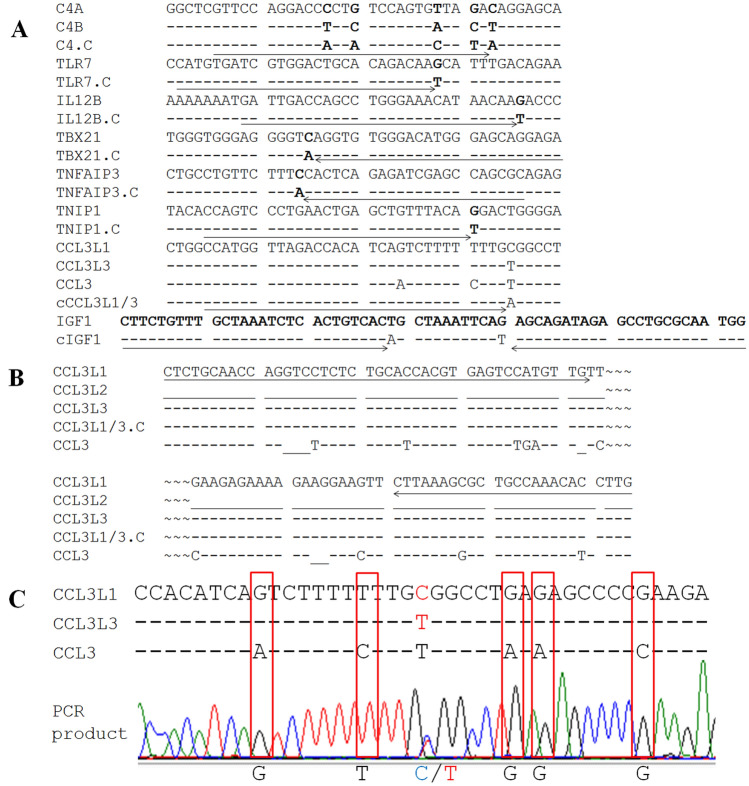
Alignments of *C4*, *TLR7*, *IL12B*, *TBX21*, *TNFAIP3*, *TNIP*, and *CCL3L* sequences for the mrcPCR assay. (**A**) Alignment of reference and competitor sequences for *C4*, *TLR7*, *IL12B*, *TBX21*, *TNFAIP3*, *TNIP*, and *IGF1*. (**B**) Alignment of reference and competitor sequences for the PCR primer sites of the *CCL3L* family. *CCL3L1*, *CCL3L2*, *CCL3L3*, and *CCL3* are *CCL3L* family genes. For (**B**) and (**C**), the same bases as those in the reference sequences are marked by “–,” the absence of a base in the sequence is marked by “_,” and the competitor sequences are named by adding ‘.C’ after the gene name. The PCR primers and extension primers for the mrcPCR assays are marked by arrows. The space sequence for the forward primers and reverse primers for the *CCL3L* family genes are marked by “~~~”. (**C**) Sanger sequencing of PCR products to confirm the specific amplification of *CCL3L1* and *CCL3L3* by the mrcPCR assay. Bases specific for *CCL3* were not observed in mrcPCR assay PCR product (red box).

The mrcPCR method, which measures CN by estimating the signals from the amplified gene products of interest relative to those from the spiked internal reference sequences, was shown to be accurate and simple for determining CNs^21,22^. To establish mrcPCR assays for *TNFAIP3*, *TNIP1*, *IL12B*, *TBX21*, *TLR7*, *C4A*, and *C4B* in the present study, modified bases were introduced to produce competitors as shown in Fig. 1.

Some inconsistencies in disease association studies of the *CCL3L* family clusters may be related to assays which provide heterogeneous results due to their designs based on incomplete information on the *CCL3L* family cluster genes^23^. To avoid these possible errors, specific PCR primers and extension primers for *CCL3L1* and *CCL3L3* were designed for the mrcPCR assay in the present study (Fig. 1B) using *IGF1* as a control gene. To confirm the specific amplification of *CCL3L1* and *CCL3L3*, Sanger sequencing of the PCR products in the mrcPCR assay was performed and confirmed that only the sequences from *CCL3L1* and *CCL3L3* were specifically amplified (Fig. 1C). This suggests that the relative CN deduced from the products in our mrcPCR assay was not confounded by other homologous sequences such as *CCL3* (geneID: 6348) and *CCL3L2* (geneID: 390788).

### Determination of CNs by mrcPCR

**Figure 2:**
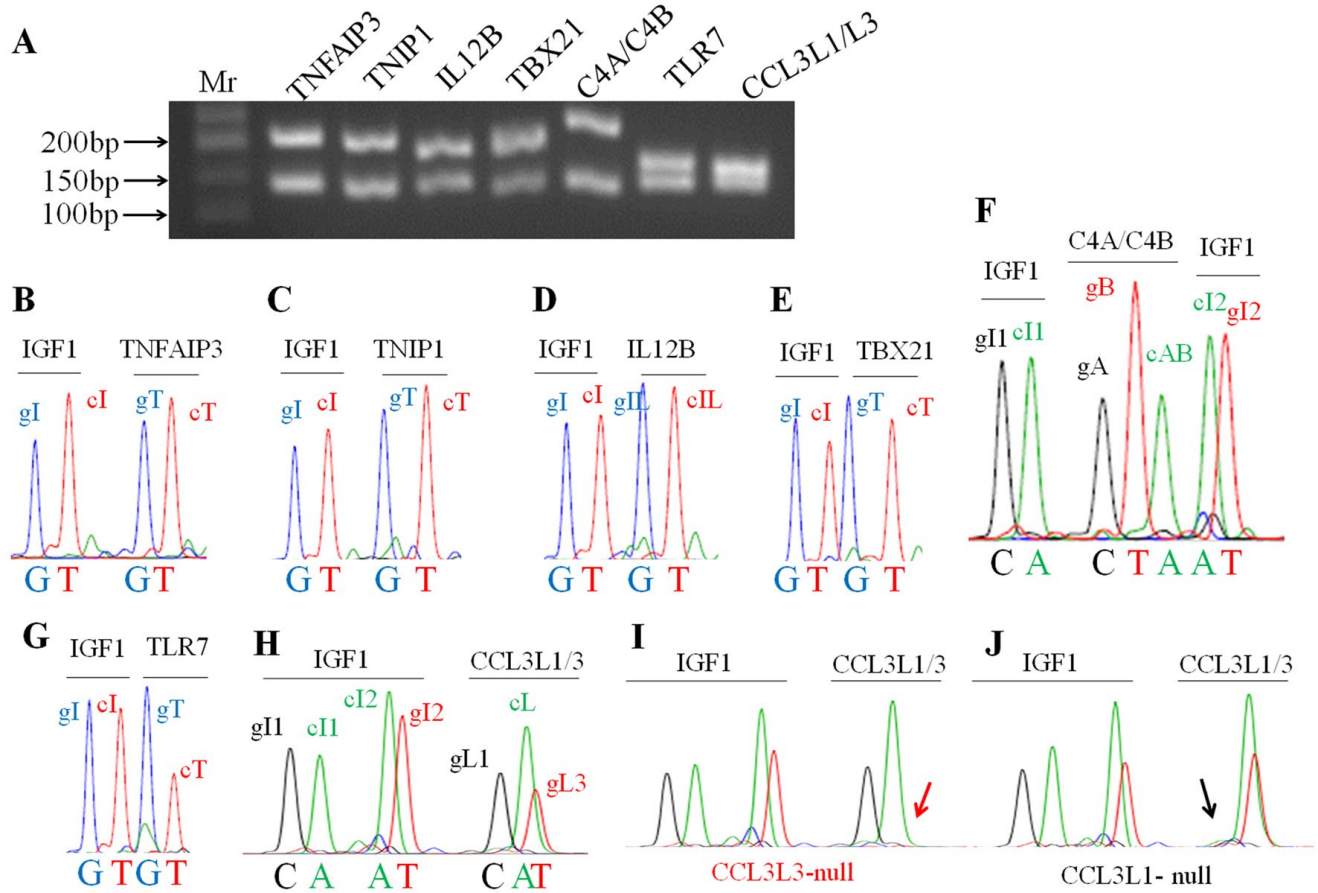
Representative results of mrcPCR assays. (**A**) Agarose gel electrophoresis for PCR products obtained in the mrcPCR assays. Target genes amplified during the mrcPCR assay are shown. *Mr.* molecular weight marker. Representative mrcPCR results for *TNFAIP3* (**B**), *TNIP1* (**C**), *IL12B* (**D**), *TBX21* (**E**), *C4A/C4B* (**F**), *TLR7* (**G**), and *CCL3L1/CCL3L3* (**H**). *CCL3L3*-null (**I**) and *CCL3L1*-null (**J**) cases are also shown. Signals from genomic and competitor sequences for the control gene, IGF1, are marked as follows: gI, gI1, and gI2 for signals from the genomic sequence; and cI, cI1, and cI2 for signals from the competitor sequence. Signals from the genomic and competitor sequences for the target genes are marked as follows: gT for signals from the genomic sequences of *TNFAIP3*, *TNIP1*, *TBX21*, and *TLR7*; gIL for signals from the genomic sequence of *IL12B*; gA, gB, gL1, and gL3 for those from the genomic sequences of *C4A*, *C4B*, *CCL3L1*, and *CCL3L3*, respectively; cAB and cL for those from the competitor sequences of *C4A/C4B*, and *CCL3L1/CCL3L3*, respectively. (**I**) Representative mrcPCR results in a *CCL3L3*-null case. (**J**) Representative mrcPCR results in a *CCL3L1*-null case. Figures (**B**) to (**J**) for mrcPCR results were obtained from GeneMapper software version 5.0 (Thermo Fisher Scientific).

**Figure 3:**
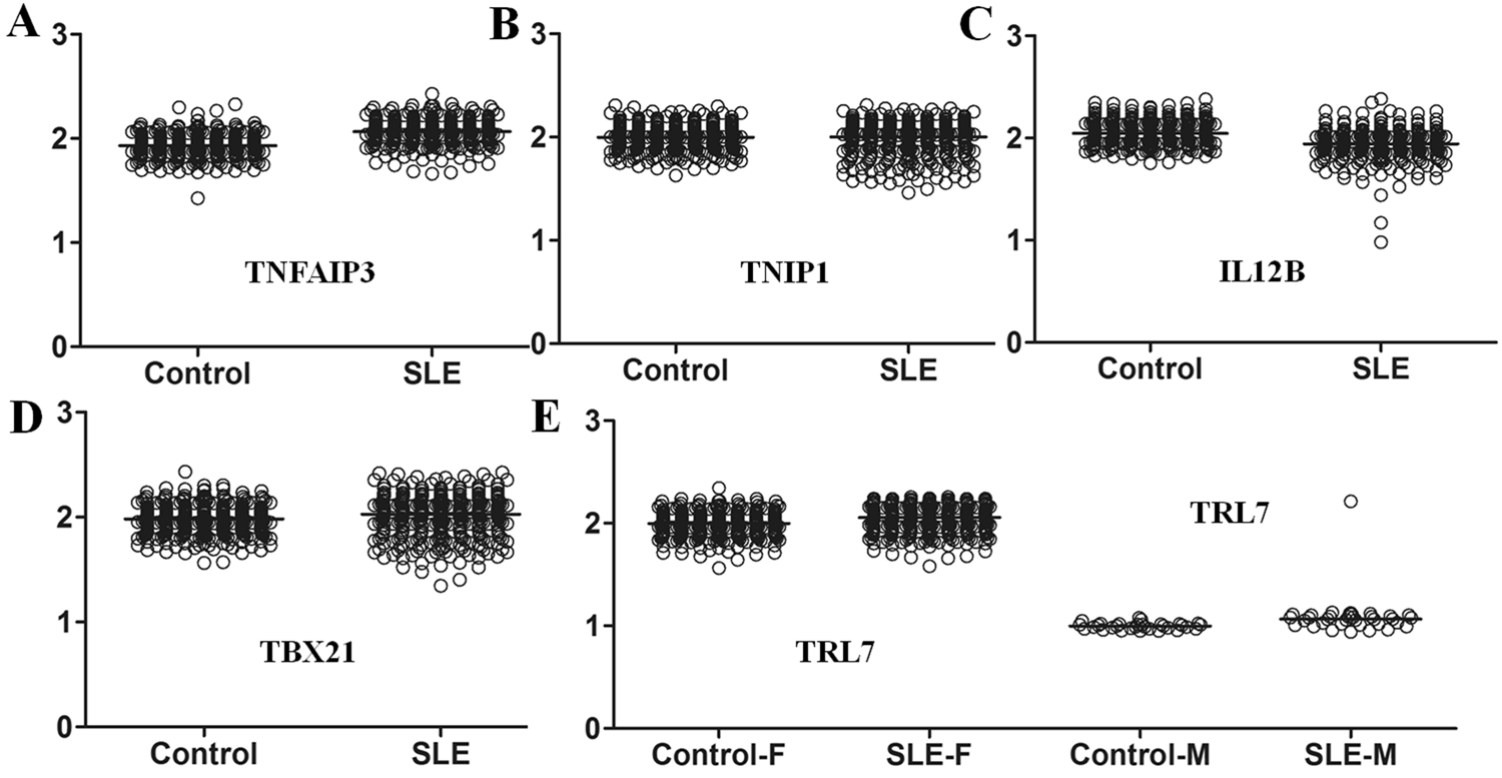
Difference in CNs between the controls and SLE patients in genes showing relatively low or no CNV. (**A**) *TNFAIP3* (*P* < 0.0001). (**B**) *TNIP1* (*P* = 0.7035). (**C**) *IL12B* (*P* < 0.0001). (**D**) *TBX21* (*P* = 0.0043). (**E**) *TLR7* (*P* < 0.0001 for both male and female). Although statistical significance was observed in several genes by the Wilcoxon rank-sum test, CNVs were not evident except in *IL12B* and *TLR7* in a few cases.

The mrcPCR assay was performed on control and SLE samples to determine the CNs of *TNFAIP3*, *TNIP1*, *IL12B*, *TBX21*, *TLR7*, *C4A*, *C4B*, *CCL3L1,* and *CCL3L3*. To optimize the assays, the relative primer concentrations and competitors were empirically determined, and the final mrcPCR assay information is shown in Supplementary Tables S1–S3. Representative mrcPCR results are shown in Fig. 2. The relative CNs were determined using the relative peak ratios from the mrcPCR results as previously reported^21,22^. After the median peak ratios values for each gene from the mrcPCR were obtained, the raw peak ratio data were divided by half of the median value, and the standardized CN (sCN) values were employed for the comparison. In genes such as *TNFAIP3*, *TNIP1*, *IL12B*, *TBX21*, and *TLR7*, CNV was absent or very low (Fig. 3). When the CNs between the controls and SLE patients were compared by Wilcoxon rank-sum tests, most genes were significant (*TNFAIP3*, *P* < 0.0001; *IL12B*, *P* < 0.0001; *TBX21*, *P* = 0.0043; and *TLR7*, *P* < 0.0001 for both male and female), except for *TNIP1* (*P* = 0.7034). However, most of the differences seemed to be related to experimental variations, as the CNs for the controls or SLE cases did not show any separate, distinct groups but showed continuous values (Fig. 3). When the CNs were compared by the Chi-squared or Fisher’s exact tests after the sCNs were transformed into digitized CNs (dCNs) as described in the Methods section, no significant CN difference between the controls and SLE cases was observed for the *TNFAIP3*, *TNIP1*, *IL12B*, *TBX21*, and *TLR7* genes (Table 1), suggesting that the direct comparison of CNs may lead to false-positive results. Although we found several distinct CNV cases in *IL12B* (Fig. 3C,E) in our dataset, the cases with CNVs were quite limited. In a male SLE patient with two copies of *TLR7* in the X chromosome, the sex was confirmed to be male by a short tandem repeat marker test, suggesting the presence of *TLR7* CN variants also in the Korean population.

**Table 1:** Correlation between gene copy number and SLE.

Gene	dCN^1^	Control		SLE		*P*^2^	Gene	dCN^1^	Control		SLE		*P*^2^
		Number	%	Number	%				Number	%	Number	%	
*TNFAIP3*	1	1	0.3	0	0	1.0000^†^	*TNIP1*	1	0	0	2	0.6	0.2414^†^
	2	337	99.7	327	100			2	338	100	325	99.4	
*IL12B*	1	0	0	3	0.9	0.1183^†^	*TBX21*	1	0	0	3	0.9	0.1183^†^
	2	338	100	324	99.1			2	338	100	324	99.1	
*TLR7*^3^ (M)	1	31	100	34	97.1	1.0000^†^	*TLR7*^4^ (F)	1	0	0	0	0	Not tested*
	2	0	0	1	2.9			2	307	100	292	100	
*C4A*	1	39	11.5	47	14.4	0.3528^‡^	*C4B*	1	43	12.7	62	19.0	**0.0379**^‡^**(0.4617)**
	2	250	74.0	242	74.0			2	223	66.0	193	59.0	
	3 or 4	49	14.5	38	11.6			3	41	12.1	51	15.6	
								4	31	9.2	21	6.4	
*CCL3L1*	0	17	5.0	18	5.5	0.6146^‡^	*CCL3L3*	0	0	0	17	**5.2**	**0.0002**^‡^**(0.0024)**
	1	98	29.0	92	28.1			1	83	24.6	74	22.8	
	2	125	37.0	134	41.0			2	141	41.7	118	36.1	
	3	56	16.6	41	12.5			3	91	26.9	84	25.7	
	4–6	42	12.4	42	12.8			4–6	23	6.8	34	10.4	
*C4B*^5^	1	43	12.7	62	19.0	**0.0331**^‡^ (0.3938)	*CCL3L3*^6^	0	0	0	17	**5.2**	**< 0.0001**^‡^**(0.0007)**
	2–4	295	87.3	265	81.0			1–6	338	100	310	94.8	

^1^dCN, digitized copy number: the standardized copy number (sCN) in which the raw copy number data were divided by half of their median value was digitized depending upon the sCN range as described in “Materials and methods”.

^2^*P*-value was estimated by the Chi-squared test (^†^) or Fisher’s exact test (^‡^). Significant differences are marked by boldface. *P*-values adjusted for multiple testing with Bonferroni’s correction method (11 tests) are shown in parentheses.

*No statistical test was performed because there was no case with a copy number of 1 for the *TLR7* gene in either the SLE patients or the controls.

^3^*TLR7* (M), *TLR7* data for males.

^4^*TLR7* (F), *TLR7* data for females.

^5^*C4B* and ^6^*CCL3L3* were analyzed for the contributions of a copy status of 1 (for *C4B*) or 0 (for *CCL3L3*) to SLE susceptibility. Odds Ratios (OR) were 1.6051 for ^5^low *C4B* CN status, and > 18.5355 for ^6^*CL3L3*-null status in SLE patients.

**Figure 4:**
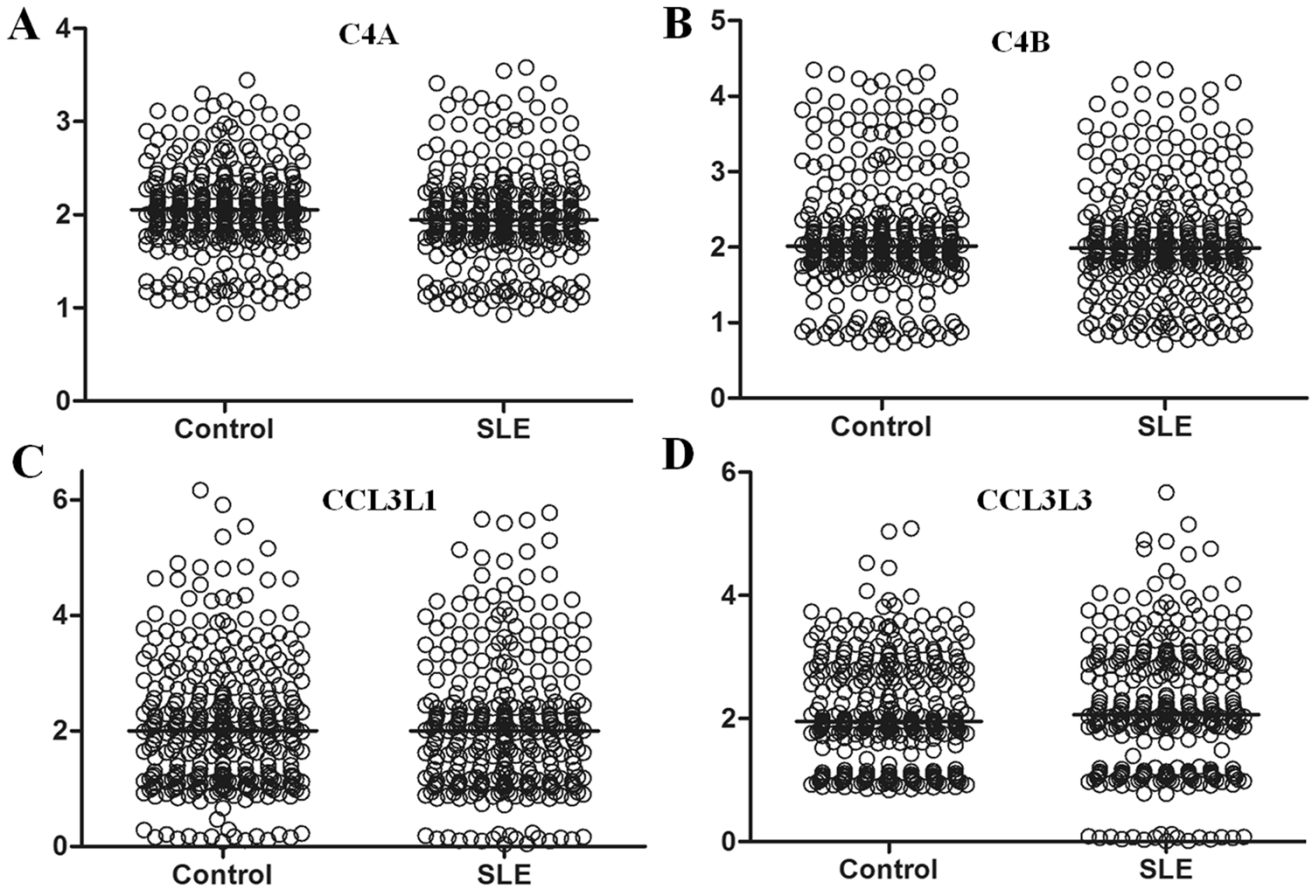
Difference in CNs between the controls and SLE patients in genes showing relatively high CNV. (**A**) *C4A* (*P* = 0.0049). (**B**) *C4B* (*P* = 0.3797). (**C**) *CCL3L1* (*P* = 0.7441). (**D**) *CCL3L3* (*P* = 0.1704). Median CN values are marked by horizontal lines.

In genes such as *C4A*, *C4B*, *CCL3L1,* and *CCL3L3*, the CNVs were relatively high (Fig. 4). In a comparison of the median CNs in those genes by the Wilcoxon rank-sum test, only *C4A* showed a significant difference (*P* = 0.0049, Fig. 4A), but the others did not (Fig. 4B–D). The significant association of CNV in *C4A* with SLE may be related to experimental variations because the significance was lost when the dCNs were compared. The dCNs were compared, *C4B* (*P* = 0.0379) and *CCL3L3* (*P* = 0.0002) CNs were significantly associated with SLE susceptibility (Table 1). Especially, the dCNs for low *C4B* (OR = 1.6051, *P* = 0.0331) and *CCL3L3*-null status (OR > 18.5355, *P* = 0.0001) were significantly associated with SLE (Table 1). After Bonferroni’s correction, the significant association between *C4B* low-copy status and SLE susceptibility became non-significant (corrected *P* = 0.3938). Therefore, only *CCL3L3*-null status, which was still significant by Bonferroni’s correction (corrected *P* = 0.0007), was considered a significant CNV for SLE in the present study.

We tested if the dCNs for *C4A*, *C4B*, *CCL3L1*, and *CCL3L3* were in Hardy–Weinberg equilibrium by the method described previously^24^. The *P*-values were significant in the controls for *C4B* (*P* = 2.0 × 10^–13^), suggesting that the dCN for *C4B* in the controls may be skewed and that the difference in *C4B* CN between the controls and SLE cases may not be valid. The *P*-values for the other cases were not significant (controls for *CCL3L3*, *P* = 0.8595; SLE cases for *CCL3L3*, *P* = 1.0; controls for *CCL3L1*, *P* = 1.0; SLE cases for *CCL3L3*, *P* = 1.0; controls for *C4A*, *P* = 0.9481; SLE cases for *C4A*, *P* = 0.9905; SLE cases for *C4B*, *P* = 0.8447).

In the analysis of associations between *CCL3L3*-null status and clinical variables, *CCL3L3*-null status showed a significant association with non-scarring alopecia (*P* = 0.0434), whereas *CCL3L1*-null status did not (*P* = 0.2048). Except for non-scarring alopecia, *CCL3L3*-null status did not show any significant association with any of the other clinical variables (Table S4).

### Confirmation of *CCL3L3*-null copy status in SLE patients by PCR sequencing and digital droplet PCR

**Figure 5:**
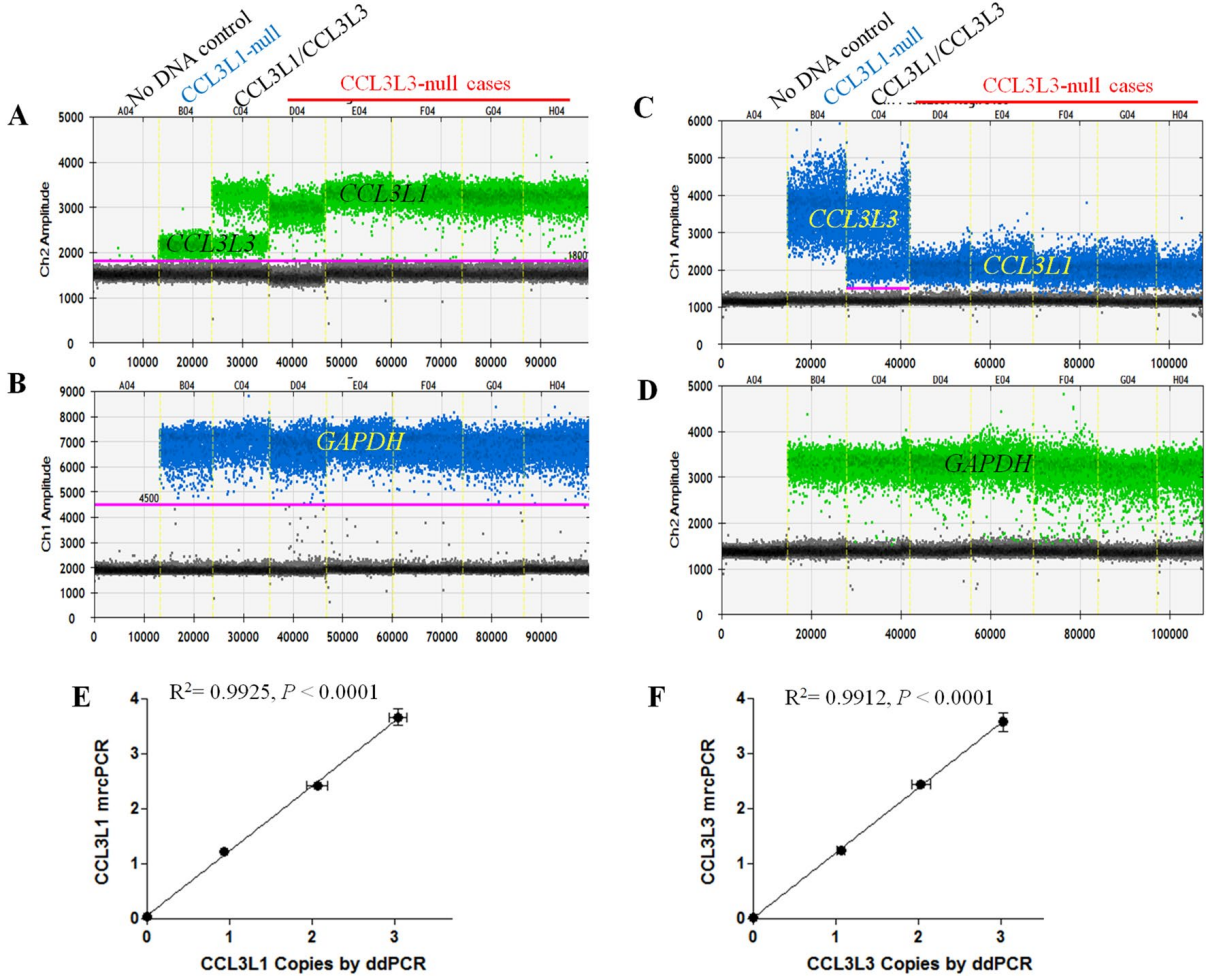
Correlation between mrcPCR and ddPCR results for *CCL3L1* and *CCL3L3*. (**A**) ddPCR results for *CCL3L1* and *CCL3L3* using a *CCL3L1*-specific probe in a *CCL3L1*-null case, five *CCL3L3*-null cases, and a case with both alleles. In addition to specific signals from *CCL3L1* (marked as *CCL3L1*), weak signals from the *CCL3L3* sequence (marked as *CCL3L3*) were also detected. (**B**) ddPCR results for the control *GAPDH* gene with a probe labeled by fluorescein phosphoramidite (FAM) in assays using a *CCL3L1*-specific probe. (**C**) ddPCR results for *CCL3L1* and *CCL3L3* using a *CCL3L3*-specific probe in the same cases as in (**A**). In addition to specific signals from *CCL3L3* (marked as *CCL3L3*), weak signals from the *CCL3L1* sequence (marked as *CCL3L1*) were also detected. (**D**) ddPCR results for the control *GAPDH* with a probe labeled by Hexachloro-Fluorescein in assays for the *CCL3L3*-specific probe in (**C**). (**E**) Linear correlation between mrcPCR and ddPCR results for *CCL3L1* (R^2^ = 0.9925, *P* < 0.0001, N = 16). (**F**) Linear correlation between mrcPCR and ddPCR results for *CCL3L3* (R^2^ = 0.9912, *P* < 0.0001, N = 16). For (**E**) and (**F**), three cases for each dCN (0–4) by mrcPCR assay were analyzed. Figures (**A**) to (**D**) for ddPCR results were obtained from QuantaSoft analysis software version 1.74 (BioRad Laboratories).

In our mrcPCR results, *CCL3L3*-null copy status showed the most significant correlation with SLE susceptibility. To confirm the *CCL3L3*-null copy status, first, we sequenced the PCR products produced in the mrcPCR assay in the *CCL3L1*-null and *CCL3L3*-null cases and found that the PCR products in the null cases did not contain the null-gene-specific base (Supplementary Figure S1), suggesting the specificity of the mrcPCR assay for *CCL3L1* and *CCL3L3*. Then, we established a digital droplet (dd)PCR assay to confirm the *CCL3L3*-null copy status in our mrcPCR results. We observed both higher and lower-level signals from *CCL3L1* or *CCL3L3* using the probe for *CCL3L1* (Fig. 5A,B), and the higher and lower level signals were switched when the probe for *CCL3L3* was employed (Fig. 5C,D), suggesting that the lower-level signals for each probe were related to cross-reactions. To evaluate *CCL3L1* and *CCL3L3* CNs by the ddPCR assay, the results from the *CCL3L1* probes were employed as the signals from *CCL3L1* and *CCL3L3* were well-separated. In the comparison of the results between mrcPCR and ddPCR, the correlations were significant (*P* < 0.0001 for both *CCL3L1* and *CCL3L3*, Fig. 5E,F), confirming that the quantitative CN results from mrcPCR were comparable to those from ddPCR. All five randomly selected *CCL3L3*-null cases from the mrcPCR assay were also negative in the ddPCR assays (five cases were shown in Fig. 5A,C), confirming again that the *CCL3L3*-null status estimated by mrcPCR was comparable to that from ddPCR.

## Discussion

Although CNVs in many immunity-related genes have implications in SLE susceptibility, the contribution of CNVs to SLE susceptibility awaits more investigation. The present study evaluated the CNs for immunity-related non-MHC genes such as *TNFAIP3*, *TNIP1*, *IL12B*, *TBX21* (*T-bet*), *TLR7*, *CCL3L1,* and *CCL3L3*, and MHC genes such as *C4A* and *C4B* using mrcPCR assays in 327 SLE patients and 338 controls. Among the CNVs for nine immunity-related genes tested in the present study, a significant correlation between *CCL3L3*-null status (*P* < 0.0001) and SLE was found.

CNVs in various non-MHC immunity-related genes including *TNFAIP3*^20,25^, *TNIP1*^20^, *IL12B*^3^, *TBX21*^3^, and *TLR7*^4,10,11^ have been reported to have an association with autoimmune diseases. Also, the correlation of CNVs in *IL12B*^3^, *TBX21*^3^, and *TLR7*^4^ with human SLE susceptibility has been reported, and the CNs over 2 were 11.3% for IL12B, 8.5% for *TBX21*, and 21.6% for *TLR7* in SLE patients in the previous reports^26,27^. However, when dCNs were analyzed in the present study, few CNVs were found in *IL12B*, *TBX21*, and *TLR7* in the SLE patients and controls, which fact may be related to ethnic differences. For *TNFAIP3* and *TNIP1*, it has already been reported that their CNVs are rare (0.2 and 0.4%, respectively)^20^, which result was reproduced in the present study. Given the few variants in those genes in the present study, therefore, we could draw any conclusions on the significance of CNVs in *TNFAIP3*, *TNIP1*, *IL12B*, *TBX21*, and *TLR7* to the SLE susceptibility of a Korean population.

*C4A* and *C4B* showed high CNV in the present study, and one copy of *C4B* (OR = 1.6051, *P* = 0.0331) was significantly associated with SLE, which is partially consistent with a previous report that suggested that one copy of *C4A* (OR = 1.613, *P* = 0.022) was a risk factor for SLE susceptibility^7^. However, we could not find *C4A*-null cases, which was suggested as a strong risk factor for SLE (OR = 5.267, *P* = 0.001) in the previous study^7^. Again, this might be related to ethnic differences. In addition, Bonferroni’s correction of the association of low *C4B* copy status lost significance (*P* = 0.3938) in the present study. In further analysis of Hardy–Weinberg Equilibrium (HWE), the CN distribution of *C4B* in the control group was not in HWE, suggesting that the comparison between the controls and cases may not be valid for the present study cases. Therefore, the correlation between *C4A* or *C4B*-null status and SLE susceptibility was not confirmed in the present study, probably due to the absence of *C4A* or *C4B*-null cases in our study cohort, which might be related to ethnic differences.

*CCL3* (geneID: 6348), *CCL3L1* (geneID: 6349), *CCL3L2* (geneID: 390788), and *CCL3L3* (geneID: 414062) are *CCL3*-related genes located closely on chromosome 17q12. *CCL3*-related genes share over 95% sequence identity at both the genomic and amino acid levels, and they encode macrophage inflammatory protein (MIP)-1α, which is secreted from epithelial cells, lymphocytes, platelets, and macrophages^26–29^. MIP-1α is a chemokine acting as a pro-inflammatory cytokine on immune cells including CD8^+^ T cells and dendritic cells via the CCR5 receptor^30^. In a previous report, SLE patients had a trend to have higher concentrations of MIP-1α and higher serum levels of MIP-1α was associated with discoid lupus^31^. Also, serum MIP-1α level was higher in patients with active renal disease than those without^32^. Lower *CCL3L1* CN was first reported to be associated with enhanced HIV/acquired immunodeficiency syndrome (AIDS) susceptibility^33^, and with the durability of immune recovery during anti-HIV-1 therapy^34^. The influence of higher *CCL3L1* CN in autoimmune diseases such as SLE^5^, rheumatoid arthritis^13^, and Kawasaki disease^35^ has been reported. However, the non-specificity of the assays used for determining the CNs in those studies due to high sequence similarity among *CCL3*-related genes has been raised, and the authors^23^ argued the necessity of new methodologies to specifically measure highly homologous *CCL3*-related genes. In addition, a report^36^ showed that the rounded *CCL3L1*-CNs were not in HWE, raising issues on the more careful interpretation of CN data.

The present study tested the association between *CCL3L1*/*CCL3L3* CNs and SLE susceptibility using an mrcPCR assay^21^, which was designed for the determination of CNs specific for *CCL3L1* and *CCL3L3*, and was not confounded by each other or the other *CCL3L*-related genes that may have confounded the estimation of CNs for *CCL3L1* or *CCL3L3* as indicated previously^23^. The specificity of our mrcPCR assay for *CCL3L1* and *CCL3L3* was confirmed by Sanger sequencing in the *CCL3L1*-null and *CCL3L3*-null cases. In addition, the distribution of *CCL3L1* and *CCL3L3* CNs by mrcPCR did not deviate from HWE in the present study, suggesting that our mrcPCR assay could be a useful tool for the validation of previous associations with susceptibility to HIV/AIDS^34^ or autoimmune diseases including RA^13^ and Kawasaki disease^35^. The significance of *CCL3L3*-null status in SLE (OR > 17, *P* < 0.0001) was maintained after Bonferroni’s correction (*P* = 0.0008). *CCL3L3*-null status by mrcPCR was confirmed by the ddPCR assay, which was developed in the present study, along with the linearity of the results obtained from the mrcPCR and ddPCR assays. Therefore, our results suggest that *CCL3L3*-null status may be a significant factor for SLE susceptibility in the Korean population.

*CCL3*-related genes encode MIP-1α which is a ligand for the CCR5 receptor. CCR5 is also the co-receptor used by the HIV-1 virus for cell entry^37,38^. Therefore, MIP-1α and the HIV-1 virus competes for the CCR5 on lymphocytes. *CCL3* and *CCL3L1* encode protein products that differ in 3 amino acids^39^, but their inhibitory effects on viral replication are tenfold different^40^, suggesting the different roles of the *CCL3*-related genes in disease susceptibility. However, *CCL3L1* and *CCL3L3* have three identical exons and encode identical proteins, and their protein products have been posited as having the same functions. Therefore, the distinction between *CCL3L1* and *CCL3L3* in their CN estimation was unimportant so far. The present study showed a significant association of *CCL3L3*-null but not *CCL3L1*-null status with SLE susceptibility, suggesting that *CCL3L1* and *CCL3L3* may have distinctive roles, and raising the question of the necessity for separate evaluation of their CNs for disease susceptibility risk assessment. Although *CCL3L1* and *CCL3L3* encode identical protein, they might have a differential role in SLE susceptibility; pertinent hypotheses, however, are not yet available. Cells from *CCL3L1*-null and *CCL3L3*-null cases may yield more insights into their possibly differential expression or roles. The present study’s significant association of *CCL3L3*-null status but not of *CCL3L1*-null status with non-scarring alopecia may also suggest their differential roles. Meanwhile, we must seek confirmation.

This study has limitations. First, the participants were ethnically limited to Korean patients, which could reduce the generalizability of our results. Secondly, this study analyzed the association between *CCL3L3*-null status and clinical manifestations of SLE based on a relatively small sample size. Further studies on various ethnic backgrounds with a larger number of SLE cases will be necessary in order to more fully explicate the significance of *CCL3L3*-null status.

## Conclusion

*CCL3L3*-null status may be a significant factor for SLE susceptibility in the Korean population.

## Materials and methods

### Patients and controls

SLE patients were recruited from a rheumatology outpatient clinic in Seoul National University Hospital. All of them had been diagnosed and followed by certified rheumatologists and met the 2019 European League Against Rheumatism and the American College of Rheumatology classification criteria^41^. The control group samples were age- and sex- matched healthy participants of Korean national health screening program. The use of samples and clinical information was approved by the Institutional Review Boards of Seoul National University Hospital and National Cancer Center and informed consent was obtained from all the participants. All methods were performed in accordance with the relevant guidelines and regulations.

### DNA isolation

DNA from blood cells was isolated using the DNeasy Blood and Tissue Kit (Qiagen, Valencia, CA, USA) and TE buffer (10 mM Tris, 1.0 mM EDTA, pH 8.0) for DNA solubilization. The purified DNA stock was maintained at − 80 °C, and diluted DNA (10 ng/μL) made from the stock using distilled water (Gibco, Carlsbad, CA, USA) was stored at − 20 °C until use.

### Cloning of competitor DNA sequences for modified real competitive PCR

To obtain the competitor sequences, the sequences for each gene were amplified with the primer pairs in Supplementary Table S1 except for *CCL3L1* or *CCL3L3,* which employed the following primers: CAA GGT GTT TGG CAG CGC TTT AAG and CTC TGC ACC ACG TGA GTC CAT GTT GTT. After purification of the amplified products and cloning into the pGEM-T Easy Vector (Promega, Madison, WI, USA), a Site-Directed Mutagenesis kit (Stratagene, La Jolla, CA, USA) was employed to introduce artificial base changes into the competitor sequences (Fig. 1A and Supplementary Figure S1). The previously reported *IGF1* competitor^21^, where two bases were changed (Fig. 1B), was employed. The cloned competitors were digested with the restriction enzyme *Sal*I to reduce non-specific amplification due to the closed circular plasmid structure. The competitors were diluted and aliquoted.

### Establishment of mrcPCR assay for the determination of CNs

PCR amplification, the purification of amplified products, primer extension reactions in the mrcPCR assay were performed as previously reported^21^. The relative peak heights for single-base-extended products were analyzed using a GeneMapper software ver. 5.0 (Thermo Fisher Scientific), and the relative CNs from the peak heights were analyzed as previously reported^21,22^. The PCR primers for mrcPCR are shown in Supplementary Table S1. For the simultaneous amplification of the genomic sequence and competitor sequence, the diluted competitor(s) was spiked into genomic DNA, and the PCR primers for the control gene, *IGF1*, were added together with the PCR primers for the gene of interest. The amount of PCR primers and the spiked competitors were determined empirically, and the amount per reaction employed in the present study is shown in Supplementary Table S2. The primers employed for the extension reaction are shown in Supplementary Table S3, along with information on the amount per extension reaction used in the present study.

The raw relative CN data for each gene were divided by half of their median value, and the resulting standardized CN (sCN) was employed for comparison by the Mann–Whitney *U* test. The sCNs were converted to digitized CNs (dCN) as follows: 0 ≤ sCN < 0.5, 0; 0.5 ≤ sCN < 1.5, 1; 1.5 ≤ sCN < 2.5, 2; 2.5 ≤ sCN < 3.5, 3; 3.5 ≤ sCN < 4.5, 4; 4.5 ≤ sCN < 5.5, 5; and 5.5 ≤ sCN < 6.5, 6.

### Droplet digital PCR for *CCL3L1* and *CCL3L3* CNs

ddPCR was carried out according to the manufacturer's protocol (QX100; BioRad Laboratories, Hercules, CA, USA). The reaction mixture was prepared according to the protocol for 2 × ddPCR Supermix (BioRad Laboratories) with 20 × primers and probes (final concentrations of 900 and 250 nM, respectively), and 25 ng of template DNA. In the reaction, the PCR amplification primers for *CCL3L1* and *CCL3L3* were the same ones employed for the mrcPCR assay (Supplementary Table S1). The detection probes for *CCL3L1* and *CCL3L3* were 5′-Hexachloro-Fluorescein (HEX)- GTC TTT TTT TGC GGC CTG AGA GC-BHQ1-3′ and 5′-FAM- GTC TTT TTT TGT GGC CTG AGA GC -BHQ1-3′, respectively. *GAPDH* was employed as a reference gene with the following primers and probe: 5′-TGC CTT CTT GCC TCT TGT CT-3′ (forward), 5′-AAT GAA GGG GTC ATT GAT GG-3′ (reverse) for amplification primers and 5′-FAM- TCA CCA GGG CTG CTT TTA AC-BHQ1-3′ (probe employed for *CCL3L1*-specific probe) or 5′-HEX-TCA CCA GGG CTG CTT TTA AC-BHQ1-3′ (probe employed for *CCL3L3*-specific probe) for the probe. Each reaction mixture was loaded into a sample well of an eight-channel disposable droplet generator cartridge (BioRad Laboratories). The emulsified samples were generated from a droplet generator (QX100; BioRad Laboratories) and then transferred into a 96-well plate. After heat-sealing with foil seal, the emulsified samples underwent a 2-step thermal cycling protocol in a T-100 Touch Thermal Cycler (BioRad Laboratories) as follows: 95 °C for 10 min, 40 cycles of 95 °C for 30 s and 55 °C for 60 s (ramp rate set to 2 °C per second), and 98 °C for 10 min. The 96-well droplet PCR plates were loaded into a droplet reader (BioRad Laboratories), which automatically read the droplets from each well of the plate. Analysis of the ddPCR data was performed with QuantaSoft analysis software version 1.74 (BioRad Laboratories).

### Statistical analyses

Age- and sex-matched SLE patients (N = 368) and controls (N = 375) were accrued. However, the cases with less than 100 ng of purified genomic DNA were excluded (N = 5 for SLE patients), and those showing failure in the mrcPCR assays for any of the seven genes (N = 36 for SLE patients, and N = 37 for controls) were excluded. Therefore, the results from 327 SLE patients and 338 controls were finally analyzed. The differences in CNs between the SLE patients and controls were evaluated by the Wilcoxon rank-sum test for continuous variables, and the Chi-squared test or Fisher’s exact test for categorical variables. The correlation of the CNs measured by ddPCR and mrcPCR was analyzed by linear regression. HWE of the CNs was evaluated by a previously reported method^24^, which estimated the expected frequencies using an estimation maximization approach and calculated the Pearson Chi-squared statistics for HWE from the expected and observed frequencies. All statistical analyses were performed using R software version 4.0.4 (R Core Team (2021). R: A language and environment for statistical computing. R foundation for Statistical Computing, Vienna, Austria. URL https://www.R-project.org/).

## Supplementary Information

Supplementary Information.
