# Supplementary material for: CCL3L3-null status is associated with susceptibility to systemic lupus erythematosus
Source: Sci Rep. 2021 Sep 27;11:19172. doi: 10.1038/s41598-021-98531-6 (PMC8476559; doi:10.1038/s41598-021-98531-6)
Supplement: Supplementary file 1 — Supplementary Information. [file 41598_2021_98531_MOESM1_ESM.docx]

**Supplementary files**

**CCL3L3-null status is associated with susceptibility to systemic lupus erythematosus**

Young-Ho Kim^a*^, Eunyoung Emily Lee^b*^, Hye-Won Sim^a^, Eun-Kyung Kang^a^, Yoon-Ho Won^a^, Dong-eun Lee^c^, Kyeong-Man Hong^a†^, Yeong-Wook Song^d,e†^

^a^Research Institute, National Cancer Center, Gyeonggi-do, Korea

^b^Division of Rheumatology, Department of Internal Medicine, Uijeongbu Eulji Medical Center, Eulji University School of Medicine, Gyeonggi-do, Korea

^c^Biostatistics Collaboration Team, Research Core Center, Research Institute, National Cancer Center, Gyeonggi-do, Korea

^d^Division of Rheumatology, Department of Internal Medicine, Seoul National University, Seoul, Korea

^e^Department of Molecular Medicine and Biopharmaceutical Sciences, Graduate School of Convergence Science and Technology and College of Medicine, Seoul National University, Seoul, Korea

*Both authors contributed equally to the study

**Supplementary Table S1**. PCR primers for amplification of competitor sequence and mrcPCR assay

| Gene | Forward (F) or Reverse (R) | Sequence | Size |
| --- | --- | --- | --- |
| C4A/C4B | F | CAGCCTTTGTGTTGAAGGTCCTGAGT | 208 |
|  | R | gggcaaagagagtcctccgacag |  |
| CCL3L1/CCL3L3 | F | GGTGTTTGGCAGCGCTTTAAGAACTTCCTT | 138 |
|  | R | CTGCACCACGTGAGTCCATGTTGTT |  |
| TLR7 | F | GCCCTGTGATGTCACTCTGGATGTTC | 150 |
|  | R | gacgctggggagatgtctggtatg |  |
| IL12B | F | AAAGCAATTTAGGGCCACTTACACTTCT | 174 |
|  | R | cagctgggaccacaagcatgag |  |
| TBX21 | F | CGTTTACCTGGTGCTGCGTCTTG | 178 |
|  | R | tgccaacgaaaaggttgaaagttactg |  |
| TNFAIP3 | F | gttcccttgctcaggcaggtaaag | 193 |
|  | R | cgcacttgggccttgaggtg |  |
| TNIP1 | F | ACTAGAAGCCACTTGCACGGTGTG | 185 |
|  | R | ccactcagcagcagggaaggagtt |  |
| IGF1 | F | TTCTCTAAATCCCTCTTCTGTTTGCTAAATC | 128 |
|  | R | gagatgggagatgttgagagcaatgt |  |

**Supplementary Table S2.** PCR component amounts for each mrcPCR assay

| mrcPCR | Components | Amount per reaction (20 μL reaction) |
| --- | --- | --- |
| C4A/C4B | C4-comp | 50 fg |
|  | IGF1-PM3-comp | 50 fg |
|  | C4 PCR primers | 5 pmole |
|  | IGF1 PCR primers | 15 pmole |
| CCL3L1/3 | CCL3L1/3-comp | 50 fg |
|  | IGF1-PM3-comp | 25 fg |
|  | CCL3L1/CCL3L3 PCR primers | 7 pmole |
|  | IGF1 PCR primers | 13 pmole |
| TLR7 | TLR7-comp | 20 fg |
|  | IGF1-PM2-comp | 50 fg |
|  | TLR7 PCR primers | 5 pmole |
|  | IGF1 PCR primers | 15 pmole |
| IL12B | IL12B-comp | 20 fg |
|  | IGF1-PM2-comp | 40 fg |
|  | IL12B PCR primers | 5 pmole |
|  | IGF1 PCR primers | 15 pmole |
| TBX21 | TBX21-comp | 20 fg |
|  | IGF1-PM2-comp | 50 fg |
|  | TBX21 | 5 pmole |
|  | IGF1 PCR primers | 15 pmole |
| TNFAIP3 | TNFAIP3-comp | 20 fg |
|  | IGF1-PM2-comp | 50 fg |
|  | TNFAIP3 PCR primers | 7 pmole |
|  | IGF1 PCR primers | 13 pmole |
| TNIP1 | TNIP1 | 20 fg |
|  | IGF1-PM2-comp | 50 fg |
|  | TNIP1 | 5 pmole |
|  | IGF1 PCR primers | 15 pmole |

**Supplementary Table S3**. Extension primers for mrcPCR assay

| Primer set | Primer ID | Extension primers | Size | Extension | **Amount (pmole) |
| --- | --- | --- | --- | --- | --- |
| C4 | C4A | TTCCAGGACCCCTGTCCAGTGTTAGA | 26 | C | 2.0 |
|  | C4B | GTTCCAGGACCtCTcTCCAGTGaTAcA | 27 | T | 1.0 |
|  | *C4.C | GGTTCCAGGACCaCTaTCCAGTGcTAtA | 28 | A | 2.0 |
|  | IGF1-1 | ccattgcgcaggctctatctgct | 23 | C/A | 0.7 |
|  | IGF1-2 | CCTCTTCTGTTTGCTAAATCTCACTGTCAC | 30 | T/A | 0.6 |
| CCL3L1/3 | CCL3L1/3 | CCATGGTTAGACCACATCAGTCTTTTTTTG | 30 | C/T/A | 1.0 |
|  | IGF1-1 | ccattgcgcaggctctatctgct | 23 | C/A | 1.0 |
|  | IGF1-3 | CTTCTGTTTGCTAAATCTCACTGTCAC | 27 | T/A | 1.0 |
| TLR7 | TLR7 | CATGTGATCGTGGACTGCACAGACAA | 26 | G/T | 1.2 |
|  | IGF1-4 | attgcgcaggctctatctgctct | 23 | G/T | 0.8 |
| IL12B | IL12B | GATTGACCAGCCTGGGAAACATAACAA | 27 | G/T | 1.2 |
|  | IGF1-4 | attgcgcaggctctatctgctct | 23 | G/T | 0.6 |
| TBX21 | TBX21 | tgatacctgctcccatgtcccacacct | 28 | G/T | 1.0 |
|  | IGF1-4 | attgcgcaggctctatctgctct | 23 | G/T | 0.8 |
| TNFAIP3 | TNFAIP3 | ttatgcgctggctcgatctctgagtg | 26 | G/T | 1.0 |
|  | IGF1-4 | attgcgcaggctctatctgctct | 23 | G/T | 0.8 |
| TNIP1 | TNIP1 | atCCAGTCCCTGAACTGAGCTGTTTACA | 28 | G/T | 1.0 |
|  | IGF1-4 | attgcgcaggctctatctgctct | 23 | G/T | 0.8 |

Extra bases (marked in red) for C4, TBX21, and TNIP1, were introduced for the interpretation of mrcPCR results by size differentiation.

*C4.C is an extension primer for the quantification of competitor C4 sequence.

** Amounts employed per extension reaction were shown.

**Supplementary Table S4.** Association of *CCL3L3*-null status with clinical variables in SLE (N = 327)

| Clinical variables |  | CCL3L3-null^†^  (N = 17) | | No null^‡^  (N = 310) | | *P*-value |  | Clinical variables |  | *CCL3L3*-null^†^  (N = 17) | | No null^‡^  (N = 310) | | *P*-value |
| --- | --- | --- | --- | --- | --- | --- | --- | --- | --- | --- | --- | --- | --- | --- |
|  |  | Number | % | Number | % |  |  |  |  | Number | % | Number | % |  |
| Gender | F | 15 | 88.2 | 280 | 90.3 | 0.6769 |  | Proteinuria | - | 6 | 35.3 | 126 | 40.6 | 0.8016 |
|  | M | 2 | 11.8 | 30 | 9.7 |  |  |  | + | 11 | 64.7 | 184 | 59.4 |  |
| Age^§^ |  | 27 (19 - 52) | | 33 (14 - 75) | | 0.6839 |  | Psychosis | - | 17 | 100.0 | 298 | 96.1 | 1.0000 |
|  |  |  |  |  |  |  |  |  | + | 0 | 0.0 | 12 | 3.9 |  |
| Malar rash | - | 8 | 47.1 | 146 | 47.1 | 1.0000 |  | Seizure | - | 15 | 88.2 | 288 | 92.9 | 0.3595 |
|  | + | 9 | 52.9 | 164 | 52.9 |  |  |  | + | 2 | 11.8 | 22 | 7.1 |  |
| Discoid rash | - | 17 | 100.0 | 282 | 91.0 | 0.3791 |  | Hemolytic Anemia | - | 10 | 58.8 | 204 | 65.8 | 0.6040 |
|  | + | 0 | 0.0 | 28 | 9.0 |  |  |  | + | 7 | 41.2 | 106 | 34.2 |  |
| Non-scarring alopecia | - | 6 | 35.3 | 189 | 61.0 | **0.0434** |  | Leukopenia | - | 7 | 41.2 | 103 | 33.2 | 0.5990 |
|  | + | 11 | 64.7 | 121 | 39.0 |  |  |  | + | 10 | 58.8 | 207 | 66.8 |  |
| *CCL3L1*-null vs  Non-scarring alopecia^††^ | - | 13 | 76.5 | 182 | 58.7 | 0.2048 |  | Thrombocytopenia | - | 13 | 76.5 | 189 | 61.0 | 0.3051 |
|  | + | 4 | 23.5 | 128 | 41.3 |  |  |  | + | 4 | 23.5 | 121 | 39.0 |  |
| Photosensitivity | - | 10 | 58.8 | 236 | 76.1 | 0.1450 |  | Lymphopenia | - | 10 | 58.8 | 143 | 46.1 | 0.3294 |
|  | + | 7 | 41.2 | 74 | 23.9 |  |  |  | + | 7 | 41.2 | 167 | 53.9 |  |
| Oro-nasal ulcer | - | 11 | 64.7 | 210 | 67.7 | 0.7941 |  | VDRL false positive^*^ | - | 17 | 100.0 | 276 | 89.0 | 0.2343 |
|  | + | 6 | 35.3 | 100 | 32.3 |  |  |  | + | 0 | 0.0 | 34 | 11.0 |  |
| Non-erosive arthritis | - | 7 | 41.2 | 118 | 38.1 | 0.8023 |  | antiDNA antibody | - | 1 | 5.9 | 34 | 11.0 | 1.0000 |
|  | + | 10 | 58.8 | 192 | 61.9 |  |  |  | + | 16 | 94.1 | 276 | 89.0 |  |
| Pleurisy | - | 14 | 82.4 | 242 | 78.1 | 1.0000 |  | Anti-Sm antibody | - | 11 | 64.7 | 230 | 74.2 | 0.4011 |
|  | + | 3 | 17.6 | 68 | 21.9 |  |  |  | + | 6 | 35.3 | 80 | 25.8 |  |
| Pericarditis | - | 15 | 88.2 | 263 | 84.8 | 1.0000 |  | ACA^**^ | - | 14 | 82.4 | 236 | 76.1 | 0.7709 |
|  | + | 2 | 11.8 | 47 | 15.2 |  |  |  | + | 3 | 17.6 | 74 | 23.9 |  |


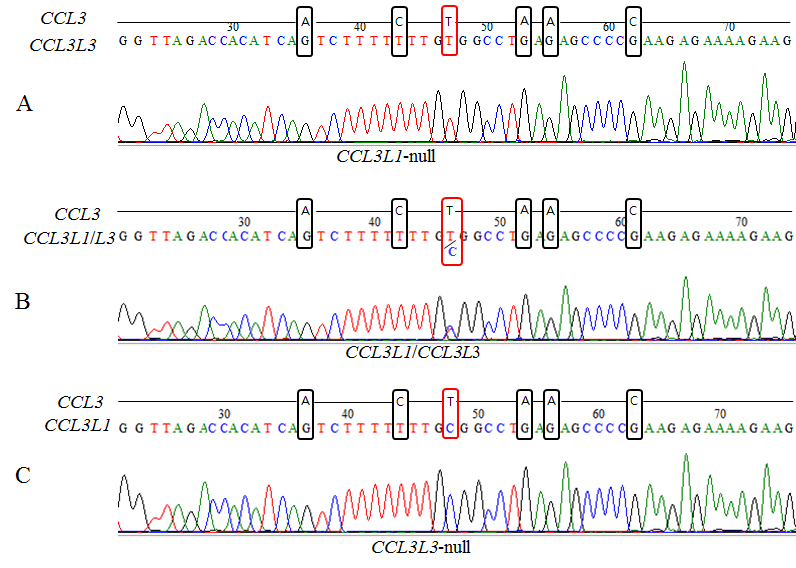


**Supplementary Figure S1**. Sanger sequencing of PCR products produced during mrcPCR assay for *CCL3L1* and *CCL3L3*. *CCL3* specific bases are marked by black square, and *CCL3L1*- or *CCL3L3*-specfic base is marked by red square. A. *CCL3L1*-null case. B. A case with both *CCL3L1* and *CCL3L3* genes. C. *CCL3L3*-null case.


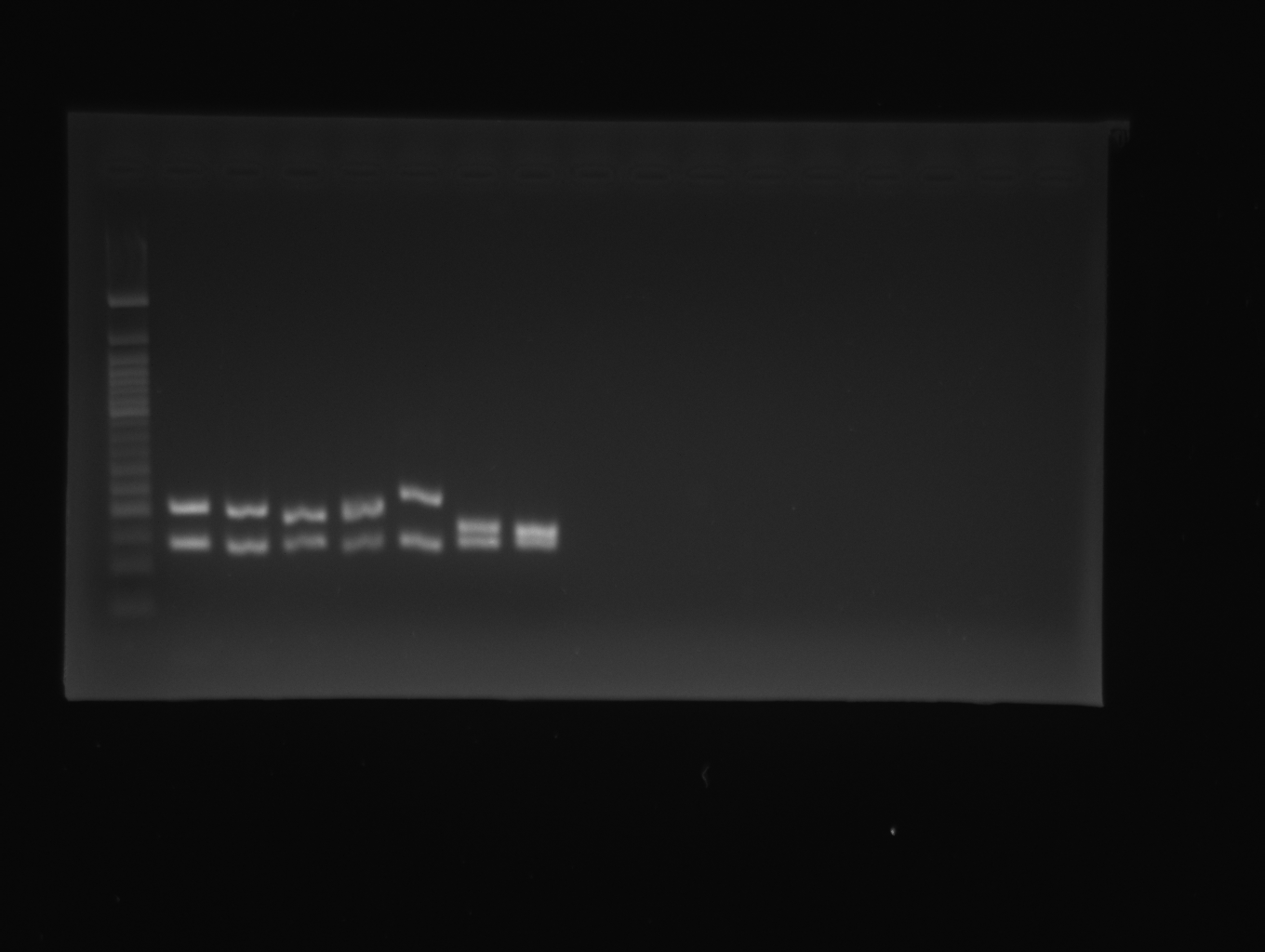


**Supplementary Figure S2.** Whole picture of agarose electrophoresis on PCR products during mrcPCR procedure in Figure 2
